# Repetitive transcranial magnetic stimulation at low frequency for the treatment of fibromyalgia. Results from the first treatment cohort at the brainwave clinic

**DOI:** 10.3389/fpain.2025.1558175

**Published:** 2025-06-25

**Authors:** Elliot Nation, Anushka Irani, Stephanie Barrett

**Affiliations:** ^1^ The Brainwave Clinic, The London Clinic, London, United Kingdom; ^2^ Department of Rheumatology, Mayo Clinic, Jacksonville, FL, United States

**Keywords:** rTMS, fibromyalgia, hypermobility, neuromodulation, rheumatology

## Abstract

**Objective:**

To assess the clinical effectiveness of low-frequency repetitive transcranial magnetic stimulation (rTMS) in treating fibromyalgia (FM) in a real-world setting.

**Methods:**

Eighteen adults diagnosed with FM received 20 sessions of low-frequency rTMS over the right dorsolateral prefrontal cortex (DLPFC). Pain and symptom burden were assessed using the Numerical Rating Scale (NRS), Fibromyalgia Impact Questionnaire (FIQ), Sheehan Disability Scale (SDS), Beck Depression Inventory (BDI), and Beck Anxiety Inventory (BAI). Outcomes were compared using paired *t*-tests.

**Results:**

Statistically significant improvements were observed in NRS, FIQ, BDI, and BAI. A non-significant trend towards reduced disability (SDS) was observed. No serious adverse effects were reported.

**Conclusion:**

Low-frequency rTMS over the DLPFC shows promise as a safe and effective treatment for FM, improving pain, mood, and impact of FM symptoms, with a trend towards improving disability. Further research with larger cohorts is needed.

## 1 Introduction

Fibromyalgia syndrome (FM) is a condition of unknown aetiology with an estimated prevalence of 3.3%–8% in the general population (1), with women being affected considerably more frequently than men (2). Typical symptoms include widespread pain, fatigue, cognitive dysfunction, sleep disturbances, depression and anxiety, all of which severely impact quality of life for people with the condition (3). Risk factors for FM include genetic predisposition, sociodemographic factors, physical inactivity, obesity, sleep disturbance, psychological factors, previous trauma (physical or emotional), hypermobility, family history, and co-existing rheumatological or autoimmune conditions (4).

Current guidance suggests initial management should focus on patient education and non-pharmacological management, with pharmacological and psychological treatments reserved as second-line options (5). The benefits of non-pharmacological treatments such as exercise therapy are also disputed. A 2017 systematic review reported that moderate-quality evidence suggests that aerobic exercise probably improves quality of life compared to control, while low-quality evidence suggests it may slightly reduce pain intensity and improve physical function, with little or no change in fatigue and stiffness (6). Inappropriately intense exercise, however, has been found to worsen symptoms in people with FM (7), so appropriate guidance is needed.

Pharmaceutical interventions, such as antidepressants and anti-epileptic drugs, are commonly prescribed, however their effectiveness in treating the condition is still a subject of debate among researchers and healthcare providers (8, 9). A 2013 meta-analysis found that while a small group of patients see notable symptom improvements from these medications, a large number discontinue treatment due to intolerable side effects, or only minimal symptom relief that is not perceived to be worth the adverse effects (10).

Cognitive behavioural therapy (CBT) is frequently recommended, with a 2018 meta-analysis finding small to moderate effect sizes in the reduction of some symptoms of FM, including depression, pain and sleep quality (11).

The primary driver of FM is thought to be central sensitisation, with studies showing abnormal descending pain modulation (4) and disrupted functional connectivity in the pain processing networks of the brains of people affected by the condition (12). Evidence to support this model includes the presence of allodynia and hyperalgesia in patients with FM, both of which are associated with nociplastic pain (13). For example, studies have shown that the stimulus necessary to elicit a pain response was almost 50% lower in FM patients than in healthy controls (14). Other studies, investigating the perceived pain response to heat, cold and electrical stimuli also found similar results, while investigations into temporal summation of pain (wind up), showed findings that are indicative of central sensitisation – including both augmentation and prolonged decay of nociception (15, 16).

Repetitive transcranial magnetic stimulation (rTMS) is a non-invasive neuromodulation technique that has been studied for its therapeutic effects in various psychiatric and neurological disorders. The mechanism of action of rTMS involves the induction of magnetic fields that penetrate the skull and induce neuronal activity in the cortex, leading to changes in cortical excitability and plasticity (17). The efficacy of rTMS is thought to depend on the frequency, intensity, and duration of stimulation, as well as the specific brain region targeted. For example, high-frequency stimulation (i.e., >5 Hz) has been shown to increase cortical excitability, while low-frequency stimulation (i.e., <1 Hz) has been shown to decrease cortical excitability (18). While these effects are short-lived, a series of treatments has been found to create long-lasting changes in both neuronal activity and cortical volume (19), highlighting the role of neuroplasticity in treatment response to rTMS.

Low-frequency stimulation of the right DLPFC is employed in rTMS treatment for fibromyalgia due to its role in modulating affective and cognitive components of pain processing via top-down control mechanisms, as well as evidence of altered right-sided prefrontal activity and inter-hemispheric imbalance in patients with fibromyalgia (20, 21). These alterations in right DLPFC function have been associated with impaired endogenous pain inhibition and heightened emotional distress, justifying targeted neuromodulation to restore functional balance and reduce symptom burden.

The use of rTMS has shown promising results as a novel treatment for FM. A 2020 randomised control trial (RCT) by Tanwar et al. found that low-frequency rTMS applied over the right dorsolateral prefrontal cortex (DLPFC) resulted in significant improvement in pain and associated symptoms in fibromyalgia patients (22). Further RCTs have supported these results, with improvements in fatigue, quality of life and fibromyalgia related mental health issues having also been found (23). The findings of these studies highlight the potential therapeutic value of rTMS as a non-invasive and effective treatment option for fibromyalgia.

Many rTMS studies to date, including the randomised controlled trial by Tanwar et al. (22), have used controlled and blinded designs. Our study contributes novel findings by evaluating low-frequency rTMS in a real-world clinical environment, providing practical insights into its use as a standard care treatment for FM. While the lack of blinding in our design limits internal validity, it improves generalisability and reflects real-world patient outcomes more accurately.

## 2 Methods

### 2.1 Participants

Patients were included if they met the following criteria: diagnosis of FM made by a consultant rheumatologist (SKB), using the 2010 American College of Rheumatology (ACR) diagnostic criteria for fibromyalgia (see Supplementary Appendix A), and over 18 years of age. Exclusion criteria were: a diagnosis of epilepsy or the presence of any metal objects in the head or neck (e.g., metal plates, clips, electrodes, stimulators, cochlear implants). All subjects were offered rTMS as a standard of care, and participation in the study was entirely optional. All patients gave informed consent for treatment before stimulation began, and were informed that treatment could be paused or stopped at any time.

Participants were also screened for Hypermobility Spectrum Disorder (HSD), including hypermobile Ehlers-Danlos Syndrome (hEDS), by SKB, using the Beighton score and 2017 international classification of the Ehlers-Danlos syndromes (see Supplementary Appendix B). Previous studies have shown an increased prevalence of FM in people with HSD/hEDS (24).

### 2.2 Intervention

All treatment was administered by a trained rTMS technician, EN at a clinic in London. Participants were first assessed by SKB to ensure that they met the inclusion criteria, before meeting EN to discuss the treatment. The initial consultation would include time for the patient to ask questions and discuss the potential benefits and risks of treatment, as well as to explore any alternative options that may be available to them.

Low-frequency (1 Hz) stimulation over the right DLPFC was chosen as the treatment protocol, based on the randomised control trial by Tanwar et al, which showed this to be effective in FM (20). A total of 1,200 pulses were delivered in each session. Stimulation was given at 100% of the patient's resting motor threshold, measured by stimulating the patient's motor cortex until a muscle twitch was elicited in their contralateral hand. This motor threshold was retested each week to ensure consistency. The Beam F3 method of neuronavigation was used to locate the right DLPFC, as this has been shown to be a reliable and accurate way to identify this target (25). Treatment was administered with a MagVenture R20 magnetic stimulator, together with a MagVenture MCF-B70 figure of eight coil, placed at a 45° angle from the midline.

Each participant underwent a total of 20 sessions in an accelerated protocol, where two sessions were delivered on each day of treatment. A 15-minute gap was left between each session. Following the final session, patients were given the option of returning for follow up sessions on a monthly basis.

### 2.3 Outcome measures

Five questionnaires were given to each patient before treatment began, and then again after six sessions, 12 sessions and 20 sessions of treatment. These included: Fibromyalgia Impact Questionnaire (FIQ), Sheehan Disability Scale (SDS), Beck Depression Inventory (BDI), Beck Anxiety Inventory (BAI), and a 0–10 Numerical Rating Scale (NRS) for pain.

### 2.4 Statistical analysis

Comparisons between pre-treatment, mid-treatment (six and 12 sessions) and post-treatment (end of 20 sessions) response was measured using paired t-tests. Statistical significance was defined as *p*-value <0.05. Analyses were completed GraphPad Prism (9.1.0).

### 2.5 Ethical approvals

rTMS is offered to patients with FM as a standard of care by SKB. All outcome measures are similarly recorded as a standard of care to assess response to therapy. No experimental endpoints were included within this study, and as per the MRC NHS REC review tool, no ethical approvals were required. All patients gave informed consent for treatment, and all data were stored confidentially in accordance with data protection regulations.

## 3 Results

In total 18 participants were included in the current study. Demographic details are highlighted in Table 1. In brief, 55% of participants were female (10/18), with a mean age of 40.89 ± 15.38 years old. Hypermobility spectrum disorders (HSD) were present in 67% (12/18) of participants, whilst hypermobile EDS (hEDS) was present in 14% (4/28). Two participants gave no history of HSD or hEDS at entry to the study. As also noted in Table 1, a number of convention pharmacological measures had been used in the previous treatment of these symptoms. Patients were advised to remain on their current medication regime for the duration of the treatment.

**Table 1:** Patient demographics.

No.	Age	Sex	Ethnicity	Diagnosis of HSD/hEDS?	Previous treatments
1	50	M	White	HSD	Duloxetine, Venlafaxine, Methylphenidate, Naltrexone
2	32	F	White	hEDS	Clonazepam
3	48	M	White	None	Duloxetine
4	27	M	White	HSD	Physiotherapy, Ibuprofen
5	39	F	White	hEDS	Amitriptyline, Naproxen
6	74	F	White	HSD	Venlafaxine
7	45	F	Asian	hEDS	Clonazepam, Diclofenac, Codydramol
8	68	F	White	HSD	Citalopram, Pregabalin, Prednisolone, HRT
9	36	F	White	HSD	Tramadol, Venlafaxine, Mirtazapine
10	41	M	White	HSD	Venlafaxine, Pregabalin, Amitriptyline
11	24	M	White	HSD	Duloxetine, Pregabalin
12	24	M	White	HSD	Venlafaxine, Sertraline
13	58	F	White	HSD	Duloxetine
14	47	F	Black	hEDS	Physiotherapy, Ibuprofen
15	25	M	White	HSD	None
16	21	F	White	HSD	None
17	48	F	White	HSD	Physiotherapy
18	29	M	White	None	Mirtazapine, Sertraline, Paroxetine

**Figure 1:**
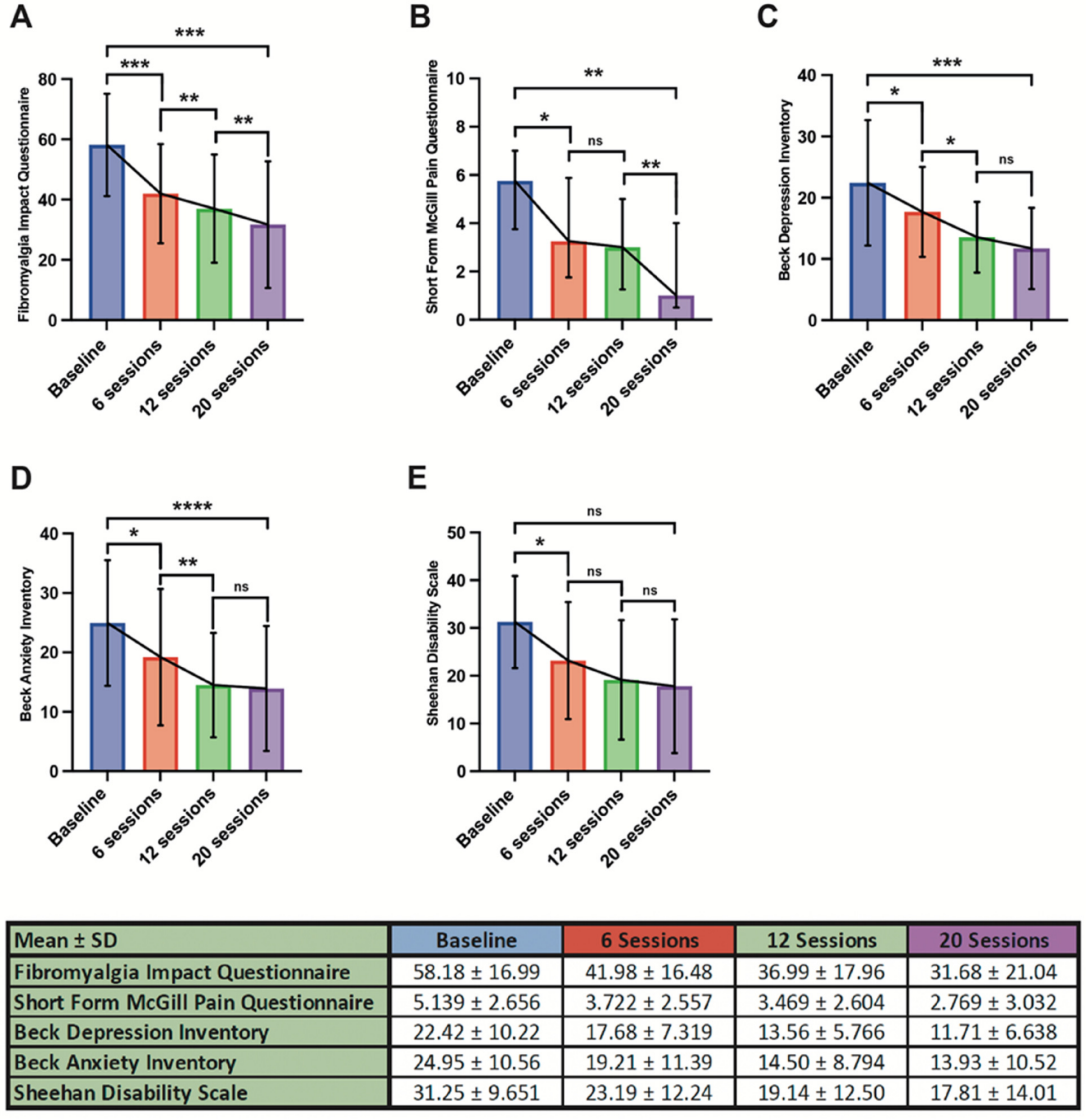
Patient responses following 6-, 12- and 20-sessions of treatment.

Mean baseline FIQ was 58.18 ± 16.99, which subsequently showed an improvement to 41.98 ± 16.48 after six treatment sessions (*p* = 0.0004). This improvement continued after subsequent sessions with a reduction to 36.99 ± 17.96 seen after 12 sessions (*p* = 0.0001) and 31.68 ± 21.04 on completing 20 sessions of therapy (*p* = 0.0001). Overall, this represented an overall mean improvement in FIQ from baseline of 26.50 after 20 sessions (*p* = 0.0001), as shown in Figure 1A.

A similar response was noted with regards to SFMPQ. At baseline this was reported as 5.139 ± 2.656 before improving to 3.722 ± 2.557 after six sessions of treatment (*p* = 0.0127). There was no significant interval improvement between 6 and 12 sessions (*p* = 0.1390), however following 20 sessions there was a noted benefit when compared with 6 sessions (2.769 ± 3.032, *p* = 0.0156). The mean difference from baseline after 20 sessions of treatment was 2.37 (*p* = 0.0039), demonstrated in Figure 1B.

Baseline BDI was 22.42 ± 10.22, which subsequently improved to 17.68 ± 7.319 after 6 sessions of treatment (*p* = 0.0292). This further improved to 13.56 ± 5.766 following a total of 12 sessions (*p* < 0.0001) but no further additive improvement was seen after 20 sessions when compared with 12 (*p* = 0.1187). Overall BDI improved to 11.71 ± 6.638 following 20 sessions, a mean improvement of 10.49 from pre-treatment baseline (*p* = 0.0002). This is summarised in Figure 1C.

In terms of anxiety, baseline BAI (24.95 ± 10.56) improved following 6 sessions of treatment (19.21 ± 11.39, *p* = 0.0219) with an ongoing improvement in BAI was seen following 12 (14.50 ± 8.794, *p* < 0.0001). As with BDI there was no additive benefit seen between 12 and 20 sessions (13.93 ± 10.52, *p* 0.4297). The total improvement from baseline was 11.02 (*p* < 0.0001), as shown in Figure 1D.

With regards to overall disability, SDS showed an initial improvement from baseline (31.25 ± 9.651) following 6 sessions of treatment (23.19 ± 12.24, *p* = 0.0389) but no further benefit was seen after a total of 12 (19.14 ± 12.50, *p* = 0.1867) and 20 sessions (17.81 ± 14.01, *p* = 0.8358). Although there was a mean improvement in SDS from baseline after 20 sessions (13.44), this did not reach statistical significance (*p* = 0.0649). This is summarised in Figure 1E.

### 3.1 Adverse events

Of the 18 participants in this study, 12 reported no adverse events or side-effects. Of those that did, two reported a short-lasting headache after treatment, while four reported a temporary increase in fatigue which lasted for up to three days. None of these side-effects persisted beyond the end of the treatment protocol. These results are in keeping with previous studies into the side-effects of rTMS which show that rTMS is a very safe and well tolerated treatment.

## 4 Discussion

The results of our study demonstrate the potential efficacy of low-frequency Repetitive Transcranial Magnetic Stimulation (rTMS) as a novel and non-invasive treatment option for Fibromyalgia (FM) patients. The primary aim of this study was to assess the impact of rTMS, specifically low-frequency stimulation over the right dorsolateral prefrontal cortex (DLPFC), on FM-related symptoms, including pain, disability, depression, anxiety, and overall quality of life. Our findings indicate significant improvements in various outcome measures, which suggest that rTMS could play a role in managing FM.

A strength of our study lies in its pragmatic design, capturing the effects of rTMS in routine clinical practice. Unlike strictly controlled and blinded trials, our results reflect outcomes that may be anticipated in broader healthcare settings. The consistency of these findings with previous blinded studies, including Tanwar et al. (22), supports the external validity of rTMS in treating FM.

One of the core findings of our study is the substantial reduction in pain and associated symptoms as measured by the Fibromyalgia Impact Questionnaire (FIQ) and the 0–10 Numerical Rating Scale (NRS) for pain. The mean improvement in FIQ from baseline was statistically significant, indicating that rTMS led to a meaningful reduction in the overall impact of FM on patients' lives. This improvement grew significantly throughout the 20 treatment sessions, suggesting a dose-dependent effect. This reduction in pain intensity, as reflected in the NRS scores, supports the potential of rTMS in alleviating one of the most debilitating aspects of FM.

The positive effects of rTMS were not limited to pain reduction alone. Our study also demonstrated significant improvements in depression and anxiety, as measured by the Beck Depression Inventory (BDI) and Beck Anxiety Inventory (BAI), respectively. These findings are particularly noteworthy, as comorbid depression and anxiety are common in FM patients and significantly contribute to their reduced quality of life. The fact that rTMS had a substantial impact on these comorbidities suggests that it could offer a holistic approach to managing FM, addressing both physical and psychological aspects.

Moreover, the reduction in overall disability, as assessed by the Sheehan Disability Scale (SDS), indicates that rTMS may improve the ability of FM patients to engage in daily activities and lead more functional lives. Although the change in SDS did not reach statistical significance after 20 sessions, the initial improvement after 6 sessions is promising, and further research could explore how to maximize this effect. A possible reason for this lack of statistical significance is the fact that reconditioning the body to a state where increased activity is possible can take weeks, or even months, after symptoms improve. Future studies with longer follow-up periods will be useful in determining whether functional improvements continue to accrue.

Our findings also reaffirm the apparent link between hypermobility spectrum disorders and FM. While this study was not designed to investigate the implications of comorbid HSD/hEDS on treatment response, future research should explore whether these conditions may modulate outcomes from neuromodulatory treatments like rTMS.

Although no statistically significant differences in outcomes were observed across sex or age demographics in our sample, the small sample size limits the generalisability of these observations.

### 4.1 Limitations

This study has several limitations. The relatively small sample size restricts the power of the statistical analysis and limits generalisability. The absence of a control group or randomisation is a key limitation, making it difficult to isolate treatment effects from placebo or other influences. This limitation is due to the real-world nature of the study and its funding constraints. Additionally, the lack of objective biomarkers or electrophysiological measures is a limitation, as all outcome measures were self-reported. Furthermore, while patients were followed up for six months, longer-term data are needed to assess the durability of the observed effects.

## 5 Conclusion

Overall, our results align with previous research, such as the study by Tanwar et al., which also demonstrated the positive effects of low-frequency rTMS on FM symptoms. The consistency of these findings suggests that rTMS could be a valuable addition to the treatment options for FM patients. Further research with larger and more diverse cohorts, as well as longer follow-up periods, will be essential to confirm and build upon these promising results. If rTMS continues to show efficacy, it may offer FM patients a non-pharmacological and non-invasive alternative that addresses the multifaceted nature of this condition, ultimately improving their quality of life.

## Data Availability

The raw data supporting the conclusions of this article will be made available by the authors, without undue reservation.
