# Supplementary material for: Repetitive transcranial magnetic stimulation at low frequency for the treatment of fibromyalgia. Results from the first treatment cohort at the brainwave clinic
Source: Front Pain Res (Lausanne). 2025 Jun 25;6:1558175. doi: 10.3389/fpain.2025.1558175 (PMC12237879; doi:10.3389/fpain.2025.1558175)
Supplement: Supplementary file 1 [file Datasheet1.pdf]

# AMERICAN COLLEGE OF RHEUMATOLOGY (ACR)
# PRELIMINARY DIAGNOSTIC CRITERIA FOR FIBROMYALGIA[1]

The information contained on this form was derived from Wolfe F, Clauw DJ, Fitzcharles M-A, et al. The American College of Rheumatology preliminary diagnostic criteria for fibromyalgia and measurement of symptom severity. *Arthritis Care Res*. 2010;62(5):600-610.

## PART 1: WIDESPREAD PAIN INDEX

### HOW TO CALCULATE THE PATIENT'S WIDESPREAD PAIN INDEX (WPI)

**1.** Using the list of 19 body areas, identify the areas where the patient felt pain over the past week. As a visual aid, front/back body diagrams are included.

— Each area identified on the list counts as 1

**2.** Total the number of body areas (the WPI score can range from 0 to 19).

**Write the patient's WPI score here:** ____________________.

**Identify the areas where the patient felt pain over the past week**

- ❑ Shoulder girdle, left
- ❑ Shoulder girdle, right
- ❑ Upper arm, left
- ❑ Upper arm, right
- ❑ Lower arm, left
- ❑ Lower arm, right
- ❑ Hip (buttock), left
- ❑ Hip (buttock), right
- ❑ Upper leg, left
- ❑ Upper leg, right
- ❑ Lower leg, left
- ❑ Lower leg, right
- ❑ Jaw, left
- ❑ Jaw, right
- ❑ Chest
- ❑ Abdomen
- ❑ Neck
- ❑ Upper back
- ❑ Lower back

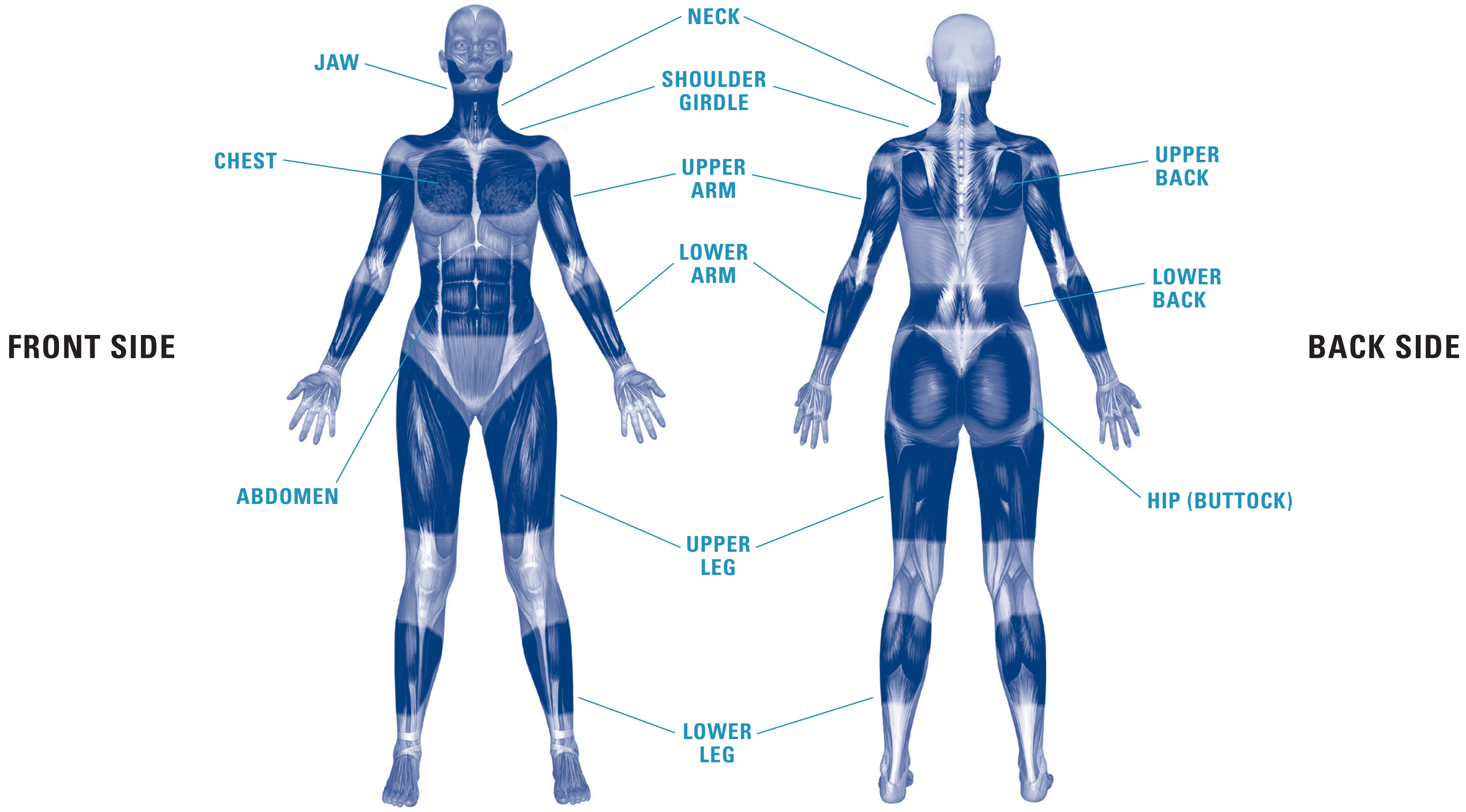

## PART 2A: SYMPTOM SEVERITY SCALE (LEVELS OF SEVERITY)

### HOW TO MEASURE THE PATIENT'S LEVEL OF SYMPTOM SEVERITY

**1.** Using a scale of 0 to 3, indicate the patient's level of symptom severity over the past week in each of the 3 symptom categories. Choose only 1 level of severity for each category.

— The score is the sum of the numbers that correspond to the severity levels identified in all 3 categories

**2.** Total the scale numbers for all the 3 categories and write the number here: ____________.

**Fatigue**

- ❑ 0 = No problem
- ❑ 1 = Slight or mild problems; generally mild or intermittent
- ❑ 2 = Moderate; considerable problems; often present and/or at a moderate level
- ❑ 3 = Severe; pervasive, continuous, life-disturbing problems

**Waking unrefreshed**

- ❑ 0 = No problem
- ❑ 1 = Slight or mild problems; generally mild or intermittent
- ❑ 2 = Moderate; considerable problems; often present and/or at a moderate level
- ❑ 3 = Severe; pervasive, continuous, life-disturbing problems

**Cognitive symptoms**

- ❑ 0 = No problem
- ❑ 1 = Slight or mild problems; generally mild or intermittent
- ❑ 2 = Moderate; considerable problems; often present and/or at a moderate level
- ❑ 3 = Severe; pervasive, continuous, life-disturbing problems

## PART 2B: SYMPTOM SEVERITY SCALE (OTHER SOMATIC SYMPTOMS)

### HOW TO DETERMINE THE EXTENT OF THE PATIENT'S OTHER SOMATIC SYMPTOMS

Using the symptoms list on the following page, determine the extent of other somatic symptoms the patient may have experienced over the past week.

**1.** Determine the quantity of somatic symptoms using the list on the following page.

**2.** Using your best judgment, calculate the score that matches the quantity of those somatic symptoms and write the number here: ____________.

**Add the scores from Parts 2a and 2b (the Symptom Severity score, or SS score, can range from 0 to 12).**
**Write the patient's SS score here: ____________.**

## OTHER SYMPTOMS

- ❑ Muscle pain
- ❑ Irritable bowel syndrome
- ❑ Fatigue/tiredness
- ❑ Thinking or memory problem
- ❑ Muscle weakness
- ❑ Headache
- ❑ Pain/cramps in abdomen
- ❑ Numbness/tingling
- ❑ Dizziness
- ❑ Insomnia
- ❑ Depression
- ❑ Constipation
- ❑ Pain in upper abdomen
- ❑ Nausea
- ❑ Nervousness
- ❑ Chest pain
- ❑ Blurred vision
- ❑ Fever
- ❑ Diarrhea
- ❑ Dry mouth
- ❑ Itching
- ❑ Wheezing
- ❑ Raynaud's
- ❑ Hives/welts
- ❑ Ringing in ears
- ❑ Vomiting
- ❑ Heartburn
- ❑ Oral ulcers
- ❑ Loss/change in taste
- ❑ Seizures
- ❑ Dry eyes
- ❑ Shortness of breath
- ❑ Loss of appetite
- ❑ Rash
- ❑ Sun sensitivity
- ❑ Hearing difficulties
- ❑ Easy bruising
- ❑ Hair loss
- ❑ Frequent urination
- ❑ Bladder spasms

**Based on the quantity of symptoms, the patient's score is:**

- ❑ **0 = No symptoms**
- ❑ **1 = Few symptoms**
- ❑ **2 = A moderate number of symptoms**
- ❑ **3 = A great deal of symptoms**

## WHAT THE PATIENT'S SCORE MEANS

**The patient's WPI score (Part 1):** __________________.

**The patient's SS score (Parts 2a and 2b):** __________________.

### A PATIENT MEETS THE DIAGNOSTIC CRITERIA FOR FIBROMYALGIA IF THE FOLLOWING 3 CONDITIONS ARE MET:

**1a.** The WPI score (Part 1) is greater than or equal to 7 and the SS score (Parts 2a and 2b) is greater than or equal to 5.

**OR**

**1b.** The WPI score (Part 1) is from 3 to 6 and the SS score (Parts 2a and 2b) is greater than or equal to 9.

**2.** Symptoms have been present at a similar level for at least 3 months.

**3.** The patient does not have a disorder that would otherwise explain the pain.

**Reference: 1.** Wolfe F, Clauw DJ, Fitzcharles M-A, et al. The American College of Rheumatology preliminary diagnostic criteria for fibromyalgia and measurement of symptom severity. *Arthritis Care Res*. 2010;62(5):600-610.

10% TOTAL RECOVERED FIBER PBP01671D1/283062-01 © 2011 Pfizer Inc. All rights reserved. Printed in USA/May 2011

Pfizer Working together for a healthier world™
