# Supplementary material for: Repetitive transcranial magnetic stimulation at low frequency for the treatment of fibromyalgia. Results from the first treatment cohort at the brainwave clinic
Source: Front Pain Res (Lausanne). 2025 Jun 25;6:1558175. doi: 10.3389/fpain.2025.1558175 (PMC12237879; doi:10.3389/fpain.2025.1558175)
Supplement: Supplementary file 2 [file Datasheet2.pdf]

The International Consortium on Ehlers-Danlos Syndromes & Related Disorders
In Association with The Ehlers-Danlos Society

Diagnostic Criteria for Hypermobile Ehlers-Danlos Syndrome (hEDS)

This diagnostic checklist is for doctors across all disciplines to be able to diagnose EDS

Distributed by The Ehlers Danlos Society

Patient name: ______________________ DOB: ____________ DOV: ____________ Evaluator: ______________

The clinical diagnosis of hypermobile EDS needs the simultaneous presence of all criteria, 1 ***and*** 2 ***and*** 3.

CRITERION 1 – Generalized Joint Hypermobility

One of the following selected:

- ☐ ≥6 pre-pubertal children and adolescents
- ☐ ≥5 pubertal men* and women* to age 50
- ☐ ≥4 men* and women* over the age of 50

Beighton Score: _____/9

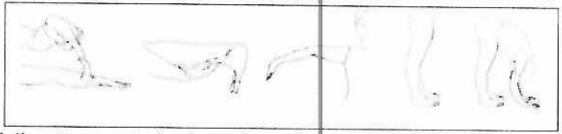

If Beighton Score is one point below age- and sex-specific cut off, two or more of the following must also be selected to meet criterion:

- ☐ Can you now (or could you ever) place your hands flat on the floor without bending your knees?
- ☐ Can you now (or could you ever) bend your thumb to touch your forearm?
- ☐ As a child, did you amuse your friends by contorting your body into strange shapes or could you do the splits?
- ☐ As a child or teenager, did your shoulder or kneecap dislocate on more than one occasion?
- ☐ Do you consider yourself "double jointed"?

CRITERION 2 – Two or more of the following features (A, B, or C) must be present

Feature A (five must be present)

- ☐ Unusually soft or velvety skin
- ☐ Mild skin hyperextensibility
- ☐ Unexplained striae distensae or rubae at the back, groins, thighs, breasts and/or abdomen in adolescents, men or pre-pubertal women without a history of significant gain or loss of body fat or weight
- ☐ Bilateral piezogenic papules of the heel
- ☐ Recurrent or multiple abdominal hernia(s)
- ☐ Atrophic scarring involving at least two sites and without the formation of truly papyraceous and/or hemosideric scars as seen in classical EDS
- ☐ Pelvic floor, rectal, and/or uterine prolapse in children, men or nulliparous women without a history of morbid obesity or other known predisposing medical condition
- ☐ Dental crowding and high or narrow palate
- ☐ Arachnodactyly, as defined in one or more of the following:
  (i) positive wrist sign (Walker sign) on both sides, (ii) positive thumb sign (Steinberg sign) on both sides
- ☐ Arm span-to-height ratio ≥1.05
- ☐ Mitral valve prolapse (MVP) mild or greater based on strict echocardiographic criteria
- ☐ Aortic root dilatation with Z-score >+2

Feature A total: _____/12

Feature B

- ☐ Positive family history; one or more first-degree relatives independently meeting the current criteria for hEDS

Feature C (must have at least one)

- ☐ Musculoskeletal pain in two or more limbs, recurring daily for at least 3 months
- ☐ Chronic, widespread pain for ≥3 months
- ☐ Recurrent joint dislocations or frank joint instability, in the absence of trauma

CRITERION 3 - All of the following prerequisites MUST be met

1. Absence of unusual skin fragility, which should prompt consideration of other types of EDS
2. Exclusion of other heritable and acquired connective tissue disorders, including autoimmune rheumatologic conditions. In patients with an acquired CTD (*e.g.* Lupus, Rheumatoid Arthritis, etc.), additional diagnosis of hEDS requires meeting both Features A and B of Criterion 2. Feature C of Criterion 2 (chronic pain and/or instability) cannot be counted toward a diagnosis of hEDS in this situation.
3. Exclusion of alternative diagnoses that may also include joint hypermobility by means of hypotonia and/or connective tissue laxity. Alternative diagnoses and diagnostic categories include, but are not limited to, neuromuscular disorders (*e.g.* Bethlem myopathy), other hereditary disorders of the connective tissue (e.g. other types of EDS, Loeys-Dietz syndrome, Marfan syndrome), and skeletal dysplasias (*e.g.* osteogenesis imperfecta). Exclusion of these considerations may be based upon history, physical examination, and/or molecular genetic testing, as indicated.

Diagnosis: ____

*Sex assigned at birth. Currently, we do not have data to provide specific additional guidance to individuals on changes in joint hypermobility following treatments that alter sex hormone levels

v9
